# Projection Mapping User Interface for Disabled People

**DOI:** 10.1155/2018/6916204

**Published:** 2018-03-01

**Authors:** Julius Gelšvartas, Rimvydas Simutis, Rytis Maskeliūnas

**Affiliations:** ^1^Automation Department, Faculty of Electrical and Electronics Engineering, Kaunas University of Technology, Studentu St. 50-154, Kaunas, Lithuania; ^2^Centre of Real Time Computer Systems, Kaunas University of Technology, Baršausko St. 59-338a, Kaunas, Lithuania

## Abstract

Difficulty in communicating is one of the key challenges for people suffering from severe motor and speech disabilities. Often such person can communicate and interact with the environment only using assistive technologies. This paper presents a multifunctional user interface designed to improve communication efficiency and person independence. The main component of this interface is a projection mapping technique used to highlight objects in the environment. Projection mapping makes it possible to create a natural augmented reality information presentation method. The user interface combines a depth sensor and a projector to create camera-projector system. We provide a detailed description of camera-projector system calibration procedure. The described system performs tabletop object detection and automatic projection mapping. Multiple user input modalities have been integrated into the multifunctional user interface. Such system can be adapted to the needs of people with various disabilities.

## 1. Introduction

People suffering from severe disabilities such as tetraplegia can also experience some speech pathologies. Such conditions drastically affect the lives of disabled people as well as their relatives. Constant care is usually inevitable, and this is both time-consuming and expensive. There is, therefore, a need for assistive technologies that can be used to perform multiple tasks. Efficient assistive technologies can improve patient's quality of life, reduce the need for care, and increase independence.

Assistive technology is a very broad term covering adaptive, rehabilitative, and assistive devices that help people perform tasks that they were formerly unable to accomplish. Assistive systems usually consist of two parts, namely, assistive devices and human computer interaction (HCI) interface. Unfortunately, most consumer grade-assistive devices are either inefficient or very expensive. Specialized user interface (UI) applications can improve assistive device efficiency and adaptability.

Each disability is unique and can change over time; therefore, the best outcomes can only be achieved by personalizing assistive technologies and UI. Majority of assistive devices have a dedicated HCI software. This makes it difficult to change devices, because the user has to learn new software. This paper presents an architecture of a multifunctional UI that can be used with multiple assistive devices. Moreover, such architecture makes it possible to use multiple devices simultaneously.

Brain computer interface (BCI) has made significant advances in recent years. Advanced BCI devices offer very natural HCI method. It has been shown in [9] that BCI systems can be used to directly control robotic arms. More recently, similar system has been used to control patient's arm [1]. These advances are, however, made with expensive and usually invasive BCI devices. Consumer-grade BCI devices are still inefficient and produce unreliable results when user is distracted [12].

Eye tracking is another technology that is advancing significantly. Moreover, affordable eye tracking devices have been shown to produce reasonable results [4]. Gaze control interfaces are already used by disabled people [14]. Eye trackers also have some limitations. The tracking can be significantly affected by head movements and sudden lighting changes.

Sip/puff switch is the most popular assistive device. This device can be easily integrated into any assistive system and is inexpensive. Sip/puff device generates two input signals that are mapped to mouse button clicks on a computer. Despite their limitations, switch devices are successfully being used for assistive and rehabilitation purposes [16].

Multifunctional UI presented in this paper can be used to perform several different tasks, namely, text input and object selection. Text input functionality uses a specialized text predictor described in [6]. Advantages of using multiple assistive devices simultaneously are presented in [5]. This paper focuses on object selection functionality of our system. Objects should be placed on a tabletop in front of the user. There are two use cases for object selection UI. First, this UI can be used by a person that is unable to speak but can use an assistive device. The user can then inform a caregiver that he wants a particular object by highlighting it with a projector. This creates a natural interaction method, because both user and caregiver see the highlighted object in the scene. Second, the selected object could be manipulated by an actuator. This would require an intelligent robotic arm that could manipulate the object when it is selected. For example, when the user selects a glass of juice, it is grabbed by a robotic arm and brought to user's mouth so that he can drink from it.

The main advantage of our object selection UI is natural HCI. Conventional UI use displays to present information for the user. In this case, the user would have to switch his attention from the monitor to the scene. Another alternative UI presentation method is using virtual reality. Severely disabled people have communication barriers, and using virtual reality headset would further separate the user from the environment. Whereas, projection mapping UI is visible not only for the system user but also by people nearby. Augmented reality has successfully been used in assistive technologies and rehabilitation [10].

The presented system consists of projector, depth camera (such as Microsoft Kinect), and at least one user action input device. Depth camera output is used to detect tabletop plane and objects positioned on that plane. Finally, projector is used together with depth camera to create a camera-projector system that performs automatic projection mapping.

Projection mapping can either be manual or automatic. Manual projection mapping is mostly used in entertainment and art industries where the scene is static. Automatic projection mapping is a more sophisticated method that works in dynamic environments. Automatic projection mapping needs 3D information of the scene obtained with depth camera. Depth camera information has to be transformed into projector optical frame. This transformation is obtained by calibrating camera-projector system. Method presented in [11] can be used when the camera is already calibrated. Alternatively structured light can be used for camera-projector calibration [13]. Both of these methods are difficult; therefore, this paper utilized a practical calibration method proposed in [22].

**Figure 1:**
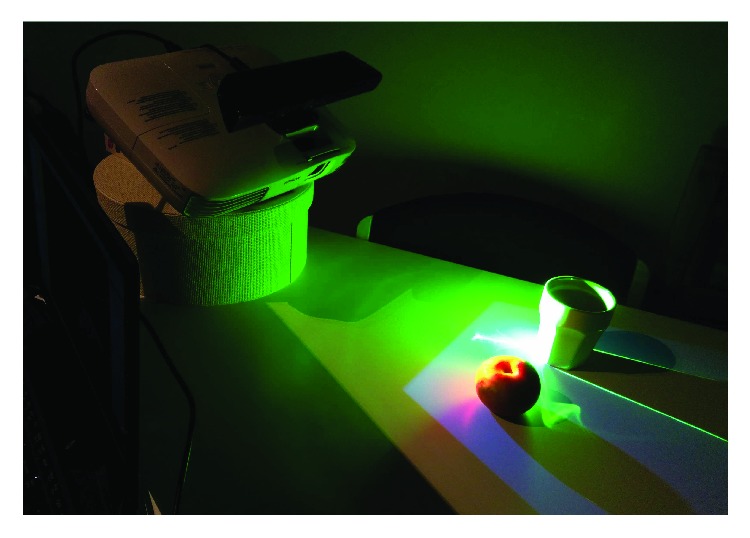
Example scene showing object selection UI that is shown to the user with camera-projector system. The system performs automatic projection mapping using depth camera information.

The system presented in this paper is similar to [2], but without the accounting for deformations caused by physical objects. Accounting for deformations is not necessary in our system, because only nonoccluded objects are detected and highlighted. More advanced dynamic projection mapping methods have been created in recent years [19]. Such systems require more expensive hardware setup.  Figure 1 shows the experimental setup of the presented system.

The remaining paper is structured as follows.  Section 2 describes the proposed projection mapping-based system. The results and discussions are presented in Section 3.  Section 4 is the conclusion.

## 2. Materials and Methods

The object selection UI highlights objects in the scene using automatic projection mapping. Automatic projection mapping can be split into three distinct stages. First, camera-projector system has to be calibrated. Calibration is a one-time procedure performed during system setup. The second stage is object detection in depth camera images. And the third stage is object highlight calculation and display using projector. Both the second and third steps are performed continuously to create a dynamically changing user interface.

### 2.1. Camera-Projector Calibration

Calibration process begins by setting up the camera-projector system. Camera and projector have to be fixed sturdily to each other. Ideally, the system should be fixed to a common metal frame or integrated into one case. Camera and projector position or orientation changes with respect to each other invalidate calibration, and the process has to be repeated. The camera-projector system position can change after calibration is finished.

**Figure 2:**
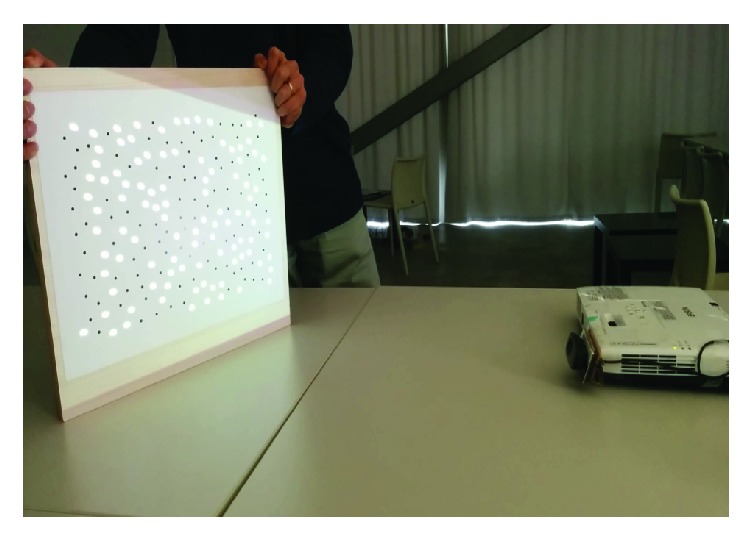
Camera-projector system calibration using a circular black dot pattern. Image was taken from [22].

Calibration is performed by placing a small calibration board in a form of camera-projector system. The board has to be stationary for one second before capturing calibration image. The system is calibrated once a predefined number of images are captured. Calibration board with random circular black dot pattern is used. During calibration, projector shows a similar white dot pattern that appears on the calibration board.  Figure 2 shows calibration procedure. Pattern detection and tracking are performed using local geometric consensus algorithm [21]. Detected point correspondences are used to estimate camera-projector system intrinsic and extrinsic parameters.

Both camera and projector are described using pinhole camera model. In this case, projector is treated as an “inverse camera.” Camera intrinsic parameters consist of two matrices, namely, camera matrix **K** and distortion coefficient matrix **D**. More precisely,

(1) $$\begin{array}{c} K=\left(\begin{array}{ccc} fx & \gamma & px\\ 0 & fy & py\\ 0 & 0 & 1 \end{array}\right), \end{array}$$

where *f*_*x*_ and *f*_*y*_ are the focal lengths, (*p*_*x*_, *p*_*y*_) is the position of the principal point, and *γ* is the camera skew. And distortion coefficients are defined as follows:

(2) $$\begin{array}{c} D=\left(\begin{array}{ccccc} k_{1} & k_{2} & p_{1} & p_{2} & k_{3} \end{array}\right), \end{array}$$

where *k*_1_, *k*_2_, *k*_3_ are the radial distortion coefficients. *p*_1_ and *p*_2_ are the tangential distortion coefficients. A more detailed description can be found in [3]. Calibration process also estimates extrinsic camera-projector system parameters, namely, a translation vector **T** and rotation matrix **R**. **T** and **R** are defined as transformation from projector optical origin to camera optical origin.

Calibration algorithm continuously detects calibration board circles. Detected circle positions are matched to circle pattern model. The camera is calibrated when sufficient number of corresponding circles is detected. Camera calibration is performed using [23] method. Camera image lens distortions are accounted for after camera is calibrated. For the projector, the board-camera homography *H*_c_b is calculated from detected circle correspondences. The projected circle locations can then be expressed as *H*_cb_^−1^*y*^(c)^ where *y*^(c)^ is the projected circle board viewed from the camera. This creates a new set of correspondences that are used to calibrate the projector and estimate camera-projector extrinsic parameters. See [22] for more detailed description of camera-projector system calibration process.

### 2.2. Object Detection

Object detection is performed on a 3D point cloud that is obtained by reprojecting each depth image pixel (*u*, *v*, *Z*) to 3D point (*X*, *Y*, *Z*, 1). Here, *u* and *v* are pixel coordinates along image rows and columns. *X* and *Y* are the 3D point coordinates along *X*- and *Y*-axis in meters, and *Z* is the point distance from the camera in meters. This transformation is performed using camera projection matrix **P**_c_. **P**_c_ is obtained by combining camera matrix **K** and distortion coefficients matrix **D** [7].

The created 3D point cloud is in the camera optical frame. Detected object coordinates should be in projector optical frame coordinates. The 3D point cloud coordinates, therefore, have to be transformed using extrinsic camera-projector system parameters. The point cloud is translated using **T** and rotated using **R**.

This paper uses tabletop object detector from *Object Recognition Kitchen* package [20]. The detector has two parts, namely, a table finder and an object recognizer. The tabletop surface is detected using RANSAC algorithm described in [15]. The tabletop plane is used to extract object points from the 3D point cloud. The extracted points are clustered into individual objects. Object type is determined by comparing object clusters to object meshes stored in the database. Exact 3D object pose is estimated using iterative closest point algorithm. Object detector output is pose and type of each detected object. This information is used to create object highlights.

### 2.3. Highlight Image Creation

The detected object models have to be rendered into a 2D image that is shown by the projector. Visualization module of the Point Cloud Library [18] was used to render object highlights. A virtual 3D scene containing a camera is created in 3D viewer. Camera parameters are chosen such that they match intrinsic parameters of the projector.

Detected object list is tracked and updated each time a new list of detected objects is received. Each object is assigned to a unique identifier. The index of the current object is also stored so that this object can be highlighted in a different color.

Each detected object is inserted into a 3D viewer using a pose obtained during object detection. Inserted object meshes are assigned a uniform color material. When all objects are inserted into the scene an image generated using a virtual camera is shown on a projector. This image projects object highlights on real scene objects.

## 3. Results and Discussion

**Figure 3:**
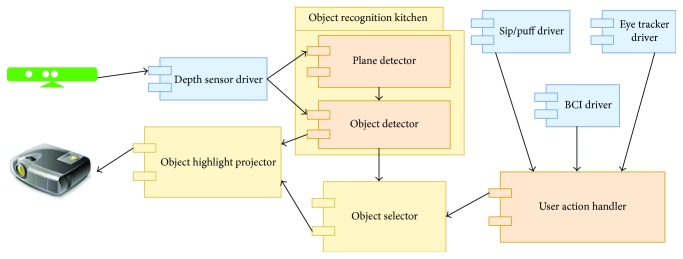
Multifunctional user interface system architecture. UI integrates multiple input modalities and a camera-projector system to perform automatic projection mapping.

The object selection functionality described in the previous section was integrated into a specialized multifunctional UI. The system architecture of this UI application can be seen in Figure 3. Figure 3 highlights the components of the multifunctional UI system.

Depth sensor driver is used to acquire depth images from Kinect senor. Note that other depth sensors can be integrated into our system. The acquired depth images are transformed to projector frame and used in object detection module. The system can use any detector that works with 3D point clouds or depth images. Current implementation used a simple tabletop object detector. A more advanced detector such as that proposed in [8] would improve detection accuracy.

User action handler is a module that handles input modality actions. This module has simple interface suited for new device integration. It is possible to configure the module to use one or multiple input modalities simultaneously. The interface supports two actions, namely, selecting current object and moving to the next object in the list. In some situations, device can only generate a single action. This problem can be solved by creating a virtual device that performs automatic moving to the next object at the given intervals. This solution is less efficient but is sometimes the only way to use the system. Current implementation supports sip/puff, eye tracking, and consumer grade BCI devices.

Object selector keeps track of all detected objects. The object list is updated each time a new depth image is processed. The update process removes objects that have been removed from the scene and adds newly appeared objects. This module also maintains an index of the current object. This index is updated by input modality actions. Finally, this package can also generate external signals, when a particular object is selected. The external signal can either be sent to the caregiver or used to initiate robotic arm actions.

Object highlight projector module implements automatic projection mapping described in the previous section. This module has access to an object mesh database. Meshes are used to generate realistic and accurate object highlights. This module also receives the current object index so that this object could be rendered in a different color.

The main advantage of described UI is that the user interacts with the environment by looking directly into the scene. Conventional UI presentation method using a display requires that the user constantly changes his attention from the display to the scene. Projection mapping also has some disadvantages. Projected light might not be visible in very bright environments, especially when the projector is not powerful. Moreover, shiny, reflective, and transparent objects can create reflection artifacts that make object highlights difficult to see. The easiest way to overcome these problems is to make sure that the scene contains only diffuse objects. The proposed system is going to be operated in a controlled environment, and the correct object choice should be performed by the system setup personnel. Alternatively, highlight visibility could be improved by projecting animated or textured highlights.

Object selection UI was evaluated using a user experience and system usability questionnaire described in [17]. Ten healthy individuals were asked to use the UI to select one of three cans placed in front of them on a tabletop surface. Each individual used the system three times with a sip/puff device for action input. After the experiment, each individual was asked to fill up the system usability questionnaire containing 16 questions. Each question was given a score from 1 to 7, where 1 means the user strongly agrees with the statement and 7 means strong disagreement. The user experience questionnaire results are summarized in Table 1. One question has been excluded from the questionnaire since it is related to system documentation and our system is only a prototype.

## 4. Conclusions

This paper presents a multifunctional UI designed specifically for people suffering from severe disabilities. The UI contains a natural augmented reality object selection HCI method. Augmented reality is achieved using automatic projection mapping. Calibrated camera-projector system is used to project object highlights. This paper describes the camera-projector system calibration setup and procedure.

The described object selection UI is intended for two use cases. First, the system can be used by people with speech pathologies. In this case, the user can highlight the objects that he wants. Without such interface, a caregiver has to constantly ask questions to find out what a disabled person wants. Second, such interface could be used in a more sophisticated setup with a robotic arm. When the user selects an object, the robotic arm can bring this object to him. In this case, the user can, for example, drink independently without the help of a caregiver.

The functionality described in this paper is part of a multifunctional UI that can be used for text entry, item, or image selection from a list and other tasks. The main advantage of a single multifunctional UI is its adaptability. The system can be configured according to the needs of individual person and adapted when his condition changes. This is achieved by integrating multiple user action input modalities.

The ideas presented in this paper could also be used with head-up display (HUD). HUD would show a more realistic object highlight and would not have problems in bright environments. Augmented reality created using HUD, on the other hand, would only be visible to the system user but not to nearby people. Available consumer grade HUD systems, however, are very expensive, whereas projectors are widespread and affordable.

The object selection interface usability has been evaluated using a specific questionnaire. Overall, the interface has been evaluated positively by the system users. Majority of users have learned how to use the system very quickly. As expected, survey relieved that the interface lacks error handling functionality. This was expected as the interface currently is a prototype and little attention has been put into error handling scenarios. These limitations will be addressed in further system developments.

## Figures and Tables

**Table 1:** Table containing user experience questionnaire and results.

(1)	Overall, I am satisfied with how easy it is to use this system.	1.8 ± 0.2
(2)	It was simple to use this system.	2.3 ± 0.8
(3)	I was able to complete the tasks and scenarios quickly using this system.	1.5 ± 1.3
(4)	I felt comfortable using this system.	4.0 ± 2.0
(5)	It was easy to learn to use this system.	2.0 ± 0.5
(6)	I believe I could become productive quickly using this system.	3.9 ± 0.7
(7)	The system gave error messages that clearly told me how to fix problems.	6.1 ± 0.9
(8)	Whenever I made a mistake using the system, I could recover easily and quickly.	5.9 ± 0.8
(9)	The information (such as online help, on-screen messages and other documentation) provided with the system was clear.	N/A
(10)	It was easy to find the information I needed.	2.1 ± 0.4
(11)	The information was effective in helping me complete the tasks and scenarios.	1.5 ± 3.1
(12)	The organization of information on the system screens was clear.	1.3 ± 0.6
(13)	The interface of this system was pleasant.	3.3 ± 2.3
(14)	I liked using the interface of this system.	2.7 ± 1.8
(15)	This system has all the functions and capabilities I expected it to have.	2.3 ± 1.3
(16)	Overall, I am satisfied with this system.	1.9 ± 0.5
